# Activation of RAF1 (c-RAF) by the Marine Alkaloid Lasonolide A Induces Rapid Premature Chromosome Condensation

**DOI:** 10.3390/md13063625

**Published:** 2015-06-05

**Authors:** Rozenn Jossé, Yong-Wei Zhang, Valentin Giroux, Arun K. Ghosh, Ji Luo, Yves Pommier

**Affiliations:** 1 Developmental Therapeutics Branch and Laboratory of Molecular Pharmacology, Center for Cancer Research, National Cancer Institute (CCR-NCI), NIH, Bethesda, MD 20892-9760, USA; E-Mails: rozenn.josse@gmail.com (R.J.); yongwei.zhang@georgetown.edu (Y-W.Z.); 2 Laboratory of Cancer Biology and Genetics, Center for Cancer Research, National Cancer Institute (CCR-NCI), NIH, Bethesda, MD 20892-9760, USA; E-Mail: valentin_giroux@hotmail.com; 3 Departments of Chemistry and Medicinal Chemistry; Purdue University; West Lafayette, IN 47907, USA; E-Mail: akghosh@purdue.edu

**Keywords:** chromosome condensation, c-raf, lasonolide A, shRNA, myosin phosphatase

## Abstract

Lasonolide A (LSA), a potent antitumor polyketide from the marine sponge, *Forcepia* sp., induces rapid and reversible protein hyperphosphorylation and premature chromosome condensation (PCC) at nanomolar concentrations independent of cyclin-dependent kinases. To identify cellular targets of LSA, we screened 2951 shRNAs targeting a pool of human kinases and phosphatases (1140 RefSeqs) to identify genes that modulate PCC in response to LSA. This led to the identification of RAF1 (C-RAF) as a mediator of LSA-induced PCC, as shRNAs against RAF1 conferred resistance to LSA. We found that LSA induced RAF1 phosphorylation on Serine 338 within minutes in human colorectal carcinoma HCT-116, ovarian carcinoma OVCAR-8, and Burkitt’s lymphoma CA46 cell lines. RAF1 depletion by siRNAs attenuated LSA-induced PCC in HCT-116 and OVCAR-8 cells. Furthermore, mouse embryonic fibroblasts (MEF) with homozygous deletion in *Raf1*, but not deletion in the related kinase *Braf*, were resistant to LSA-induced PCC. Complementation of *Raf1*^−/−^ MEFs with wild-type human RAF1, but not with kinase-dead RAF1 mutant, restored LSA-induced PCC. Finally, the Raf inhibitor sorafenib, but not the MEK inhibitor AZD6244, effectively suppressed LSA-induced PCC. Our findings implicate a previously unknown, MAPK-independent role of RAF1 in chromatin condensation and potent activation of this pathway by LSA.

## 1. Introduction

Natural products stand out by the diversity of their chemical structures and by the fact that many of them act as natural toxins that bind with high selectivity to biological targets involved in vital cellular processes. For this reason, natural products represent a unique source for identifying new chemicals to probe cellular function and for discovering new therapeutic agents. Marine natural products have proven to be a vast source of highly diverse and specific anticancer drugs, as exemplified by cytarabine, trabectedin, eribulin, and the monomethylauristatin antibody-drug conjugate, as well as byzalypsis, aplidin, kahalalide and bryostatin, which are in clinical trials [1,2,3,4,5]. Natural products in general exhibit a broad range of molecular mechanisms of action including allosteric and interfacial inhibition [6,7].

**Figure 1:**
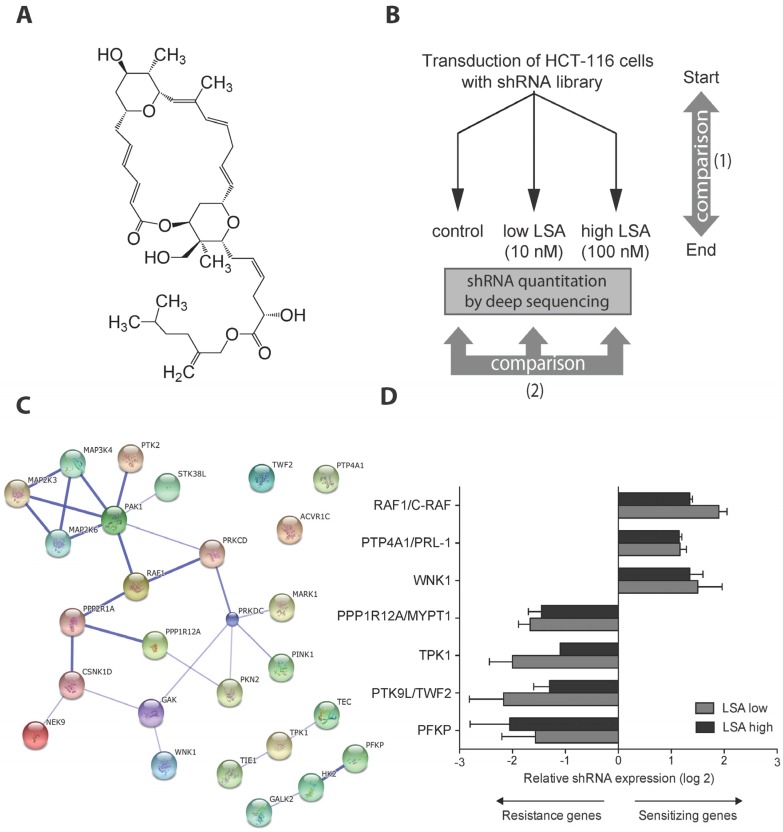
shRNA screen for genetic determinants of lasonolide A (LSA) sensitivity. (**A**) Chemical structure of LSA; (**B**) Scheme of the shRNA screen aiming to identify genes that modulate LSA sensitivity. A pooled retroviral shRNA library directed against kinases and phosphatases was transduced into HCT-116 colorectal cancer cells. Cells were cultured in the absence (control) or presence of LSA at two concentrations, 10 nM for 24 days or 100 nM for 14 days, respectively. Library compositions in starting and end samples were deconvoluted by deep sequencing to identify shRNAs that selectively dropped out or enriched in the LSA-treated samples; (**C**) Top candidates are enriched in known protein-protein interactions as determined by STRING; (**D**) shRNAs that enriched and dropped out selectively in the LSA treated samples. Results are expressed as the difference between the log2 ratio of (LSA/start) and the log2 ratio of (control/start). LSA low and high indicates the 10 nM and 100 nM treatment conditions, respectively. Genes whose shRNAs enriched with LSA treatment are potentially associated with LSA sensitivity, while genes whose shRNAs depleted with LSA treatment are potentially associated with resistance.

Lasonolide A (LSA) (Figure 1A), which was first extracted from the Caribbean marine sponge *Forcepia* sp., was noted for its high potency and unique profile of cytotoxic activity in the National Cancer Institute cell screen panel (the NCI60) [8]. The anticancer potential of LSA has remained relatively unexplored at least in part because of its low chemical abundance and challenging chemical synthesis due to its complex chemical structure comprising multiple chiral centers (Figure 1A) [9,10,11]. However, its biological effects are remarkable. LSA has rapid and profound effects on critical aspects of cell biology including inhibition of cell adhesion, activation of PKC, and activation of the mitogen-activated protein kinases (MAPK) ERK1/2 and p38 [8,12,13].

We recently showed that a major cellular effect of LSA is the induction of premature chromosome condensation (PCC) [13]. LSA-induced PCC is observed independently of cell cycle phases and is rapidly reversible upon drug removal, suggesting the selective targeting of cellular components by LSA [13]. In contrast, the cytotoxicity of LSA is dependent on drug exposure time [12,13]. During normal cell cycle, chromosomes condense in a highly ordered manner at the onset of mitosis, and PCC is defined by its occurrence outside of mitosis before completion of DNA replication. PCC can be induced by cell fusion between interphase and mitotic cells, or by the action of chemicals, primarily phosphatase inhibitors such as calyculin A or okadaic acid [14]. In contrast to okadaic acid, which requires the activation of MPF (mitosis promoting factor) to induce PCC, LSA induces PCC at nanomolar concentrations independently of MPF [13]. LSA-induced PCC is accompanied by epigenetic modifications including hyperphosphorylation and deacetylation of histones, as well as activation of topoisomerase II and aurora A/B [13]. However, the pathways that lead to such activations have not been examined.

To elucidate the molecular target(s) of LSA and identify its mechanisms of action, we undertook a shRNA screening approach using a pooled library against kinases and phosphatases (1140 RefSeqs) [15]. This led to the identification of RAF1, a serine/threonine protein kinase that plays a key role in the Ras/MAPK signaling pathway. There are three RAF kinases in human, A-RAF, B-RAF and RAF1 (C-RAF) that are believed to play non-redundant roles. RAF kinases interact with activated Ras, which recruits RAF to the plasma membrane. This results in a multistep mechanism that activates RAF, and in the case of RAF1 it involves the phosphorylation of RAF1 on serine 338 and tyrosine 341 [16]. It has been shown that S338 phosphorylation on RAF1 is carried out via autophosphorylation and via phosphorylation by PAKs (p21-activated kinases) [17,18,19,20]. RAF kinases, in turn, activate the MEK/ERK cascade, which was recently found to be rapidly activated by LSA [12]. Although MEK kinases have been classically defined as the only RAF1 substrate, it is unclear whether RAF1 has MEK-independent kinase function. Our work demonstrates rapid and reversible RAF1 activation by LSA, and its implications in the induction of PCC by LSA.

## 2. Results and Discussion

### 2.1. A Kinome shRNA Screen Identified Candidate LSA Molecular Targets and Pathways

To probe the genetic pathways that might mediate the cellular response to LSA, we screened a pool of 2951 retroviral shRNA targeting most kinases and phosphatases (1140 RefSeq transcripts) at two different drug concentrations of 10 and 100 nM in the colorectal cancer cell line HCT-116. These concentrations were previously shown to efficiently induce PCC [13]. The low and high LSA concentration allowed 10 and 4 cell population doublings during the 24 and 14 days of drug treatment, respectively. At the end of the LSA exposures, genomic DNA was extracted and shRNA library compositions in all samples were analyzed by sequencing [15]. Analysis of the differential abundance of shRNAs in cells selected after exposure to LSA compared to control cells allowed the identification of genes that are potentially involved in LSA response (Figure 1B).

From the 45 and 35 shRNA differentially enriched/depleted after exposure to the low and the high LSA concentration, respectively (Supplementary Figure S1), STRING analysis was performed to reveal protein-protein interactions among candidates identified after exposure to either low (10 nM) or high (100 nM) LSA concentration (Figure 1C). This analysis identified a pathway related to protein regulation by phosphorylation and to cell cycle and survival (GO biological processes). Moreover, among the seven candidate genes identified as having significant association with LSA response (Figure 1D), three of them were found in this pathway: RAF1, PPP1R12A, and WNK1.

RAF1, WNK1, as well as PTP4A1 were defined as sensitizing genes, as their shRNAs were enriched in the LSA-treated cells (Figure 1C). PTP4A1, also known as PRL-1 (phosphatase of regenerating liver), is a protein tyrosine phosphatase involved in cell adhesion and migration [21]. WNKs (Protein Kinase Lysine-Deficient) proteins primarily regulate ion balance; they also interact with mitotic spindles [22,23].

The four resistance genes, with corresponding shRNAs significantly depleted in the LSA-treated cells were PPP1R12A, TPK1, PTK9L, and PFKP. PPP1R12A, known as MYPT1 (Myosin Phosphatase-Targeting Subunit 1) encodes a subunit of the phosphatase PP1c that controls the actin-myosin contractile system [24]. PTK9L, also known as Twinfilin-2 (TWF2) interacts with protein kinase C-zeta and is involved in motile and morphological processes [25]. PFKP and TPK1 play a role in energy metabolism.

### 2.2. LSA Rapidly Activates RAF1 by Inducing Its Phosphorylation on Ser338

**Figure 2:**
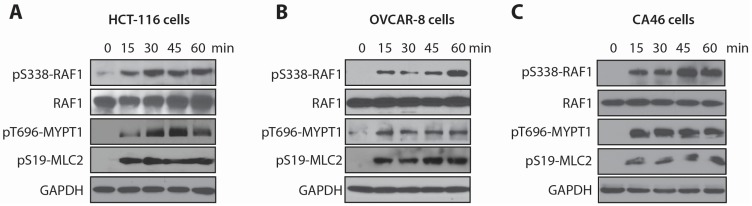
RAF1 and MYPT1, two candidates identified in the shRNA screen, are rapidly phosphorylated in response to LSA. (**A**–**C**) Time course induction of RAF1 and MYPT1 phosphorylation by LSA in HCT-116 colorectal cancer cells (**A**), OVCAR-8 ovarian cancer cells (**B**) and CA46 Burkitt’s lymphoma cells (**C**). Cells were exposed to 100 nM LSA for the indicated times. RAF1 activation was evaluated by its phosphorylation on S338. MYPT1 inhibition was evaluated by its inhibitory phosphorylation on T696 and by MLC2 phosphorylation on S19 due to its MYPT1-mediated dephosphorylation on Ser19.

Phosphorylation of S338 plays a pivotal role for RAF1 activation [16,26]. To determine the role of RAF1 in the cellular effects of LSA, we measured the kinetics of RAF1 phosphorylation on S338 in three cell lines (Figure 2). These include the HCT-116 cell line we used for the shRNA screen, the ovarian cancer cell line OVCAR-8, which is wild-type for the RAS pathway [27], and the Burkitt’s lymphoma cell line CA46, which we used in our initial study of LSA-induced PCC [13]. In all three-cell lines, LSA induced a marked phosphorylation of RAF1 within 15 min (Figure 2).

In addition to RAF1, we also measured the effects of LSA on the phosphatase MYPT1 (also known as PPP12R1A), one of the LSA resistance genes (see Figure 1C; Supplementary Figure S1). MYPT1 inhibits actomyosin contractility by dephosphorylating MLC2 (myosin light chain) on Serine 19 [28]. MYPT1 is downstream from the Rho-ROCK pathway, which phosphorylates MYPT1 on threonine 696, leading to its inactivation [29]. LSA effectively inhibits MYPT1 phosphatase activity through stimulating its phosphorylation on T696 in the three cell lines within 15 min. Consistent with MYPT1 inactivation, MCL2 phosphorylation on S19 was elevated in these cell lines (Figure 2). Together these results demonstrate that LSA engages at least two of the pathways identified in our shRNA screen within minutes of drug exposure. For rest of the study we focused on investigating the role of RAF1 in LSA induced PCC.

### 2.3. RAF1 Kinase Activity Is Involved in LSA-Induced PCC

**Figure 3:**
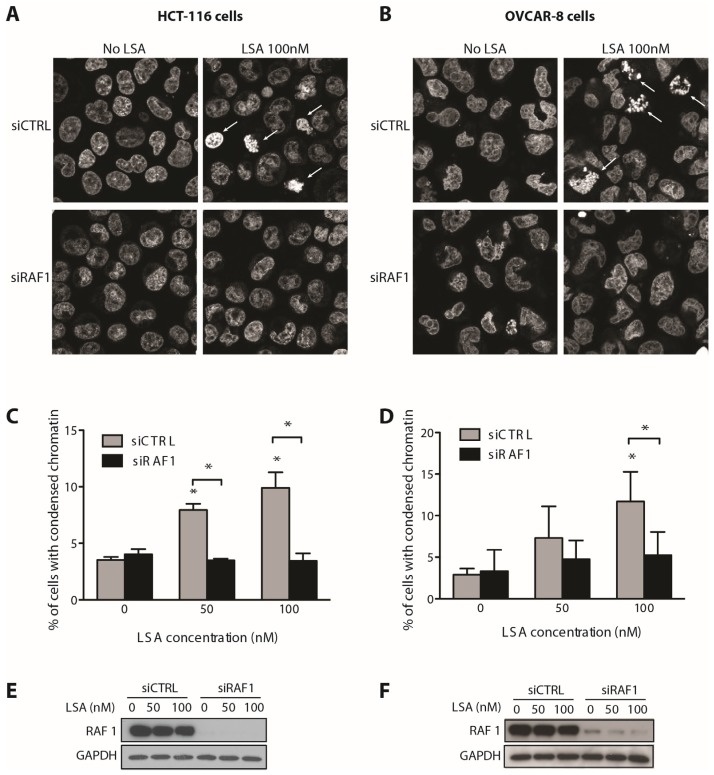
RAF1 is functionally required for LSA-induced premature chromosome condensation (PCC). (**A**,**B**) Representative images of PCC induced by LSA (100 nM, 30 min) in HCT-116 (**A**) and OVCAR-8 (**B**) cells. Cells were transduced with control or RAF1 siRNA 3 days prior to LSA treatment (100 nM, 30 min). Arrows indicate cells with PCC. DNA is visualized using propidium iodide; (**C**,**D**) Quantification of PCC induced by LSA (50 or 100 nM, 30 min) in HCT-116 (**C**) and OVCAR-8 (**D**) cells (results are mean ± SD of 3 independent experiments. * *p* < 0.01); (**E**,**F**) Immunoblots confirming knockdown of RAF1 in HCT-116 (**E**) and OVCAR-8 (**F**) cells.

To test the implication of RAF1 in LSA-induced PCC, we first knocked down RAF1 with siRNA and analyzed its impact on cellular response to LSA. The induction of PCC by LSA was markedly attenuated upon RAF1 depletion in both HCT-116 and OVCAR-8 cells (Figure 3A,B). Quantitative analysis indicated that RAF1 depletion nearly abolished LSA-induced PCC in both cell lines (Figure 3C,D). The effectiveness of RAF1 knockdown in HCT-116 and OVCAR-8 was verified by Western blotting (Figure 3E,F).

**Figure 4:**
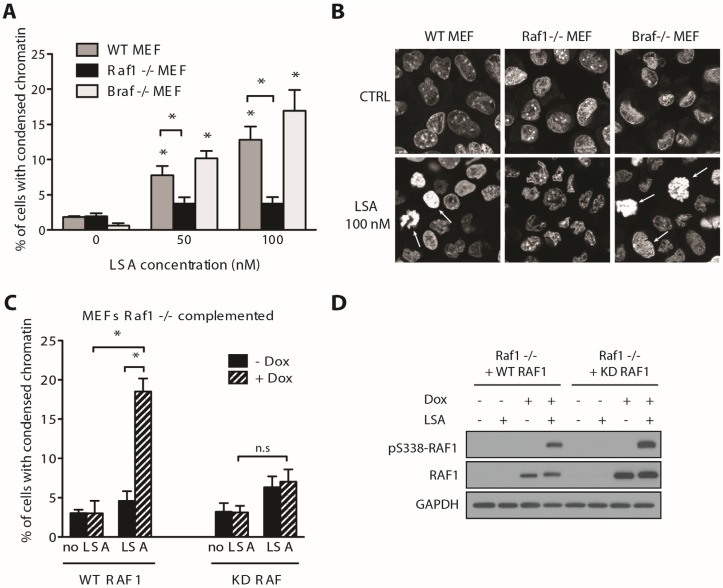
LSA-induced PCC requires RAF1 kinase activity but is not dependent on BRAF. (**A**) Relative PCC induced by LSA (100 nM, 30 min) in WT, *Raf1*^−/−^ and *Braf*^−/−^ mouse embryonic fibroblasts (MEFs). Results are the mean ± SD of 3 independent experiments. * *p* < 0.01; (**B**) Representative images of PCC induced by LSA in WT, *Raf1*^−/−^ and *Braf*^−/−^ MEFs. Arrows indicate cells counted as PCC. DNA is visualized using propidium iodide; (**C**) Chromatin condensation induced by LSA (100 nM, 30 min) in *Raf1*^−/−^ MEFs complemented with tetracycline-inducible WT or kinase dead (KD) human RAF1 cDNA. Cells were induced with doxycycline (Dox, 1 μg/mL for 24 h) prior to LSA treatment. Results are mean ± SD of 3 independent experiments. * *p* < 0.01; (**D**) Immunoblotting confirming the induction of WT or KD RAF1 by doxycycline.

Recent studies indicate that RAF1 activation requires its dimerization with BRAF [30,31]. Of note, we did not recover BRAF from our shRNA screen as a LSA sensitizing gene. To confirm that RAF1 is required for LSA-induced PCC and determine whether BRAF also plays a role in LSA induced PCC, mouse embryonic fibroblasts (MEF) that are either wild type (WT) or knockout for *Raf1* or *Braf* were exposed to LSA for 30 min. LSA induced PCC to a similar extent in both WT and *Braf^−/−^* MEFs. By contrast, LSA was unable to induce PCC in *Raf1^−/−^* MEFs (Figure 4A,B). To test whether the kinase activity of RAF1 is required for LSA-induced PCC, *Raf1^−/−^* MEFs were complemented with either a Tet-inducible wildtype human RAF1 (WT RAF1) cDNA or a Tet-inducible kinase-dead human RAF1 (KD RAF1) that carries a K375A point mutation in its kinase domain [32]. Doxycycline induction of RAF1 and its S338 phosphorylation by LSA treatment was observed for both WT and KD RAF1 (Figure 4D). However, only WT, but not KD RAF1, restored PCC in response to LSA (Figure 4C). This result shows that RAF1 kinase activity is necessary for LSA-induced PCC.

**Figure 5:**
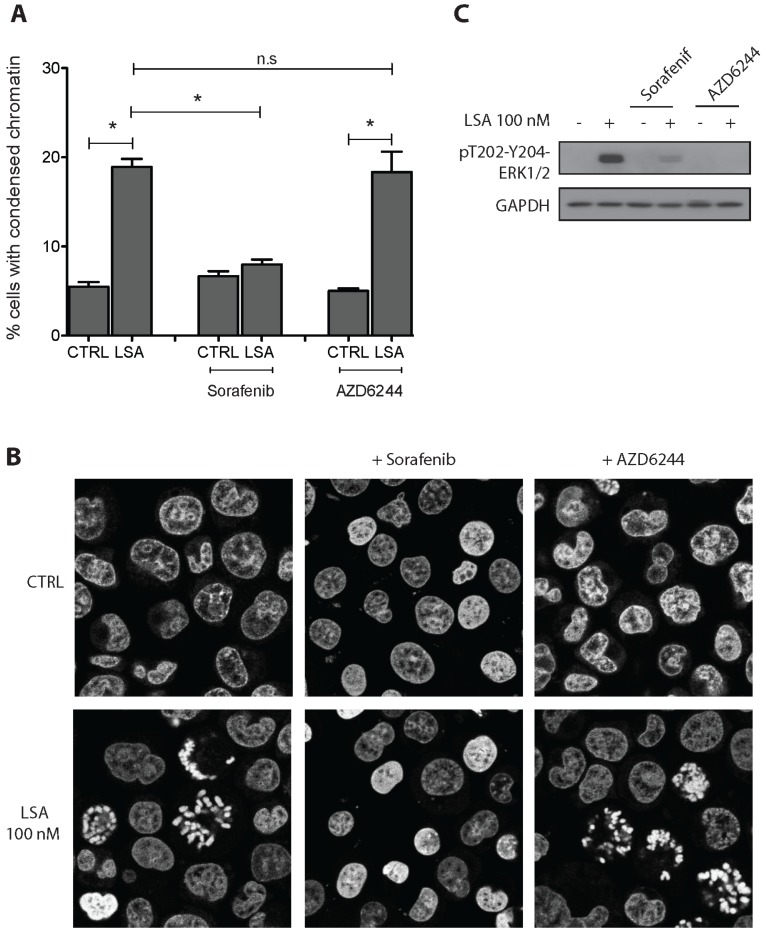
Pharmacological inhibition of RAF1, but not MEK1/2, reduced LSA-induced PCC. (**A**) PCC induced by LSA (100 nM) in HCT-116 cells pre-treated for 1 h with 10 μM of sorafenib or AZD6244 and then co-treated with LSA for 30 min. Results are the mean ± SD of 3 independent experiments. * *p* < 0.01; (**B**) Representative images of PCC induced by LSA with or without pre-treatment with sorafenib or AZD6244. DNA is visualized using propidium iodide; (**C**) Immunoblotting confirming the inhibition of ERK1/2 phosphorylation by AZD6244.

To further demonstrate that RAF1 kinase activity is critical in this context, we employed a pharmacological inhibitor of RAF1, sorafenib [33]. Treatment with sorafenib reduced LSA-induced PCC in HCT-116 cells (Figure 5A–C). Since a major effector of RAF1 is the MEK/ERK cascade, we tested whether inhibition of MEK by the small molecular inhibitor AZD6244 [34] would have the same effects. Consistent with the ability of LSA to activate RAF1, ERK phosphorylation is also strongly elevated in cells treated with LSA. To our surprise, however, AZD6244 failed to inhibit PCC induced by LSA, despite that it effectively abolished ERK1/2 activation under the same condition (Figure 5A–C). These experiments therefore demonstrate that LSA induces PCC through a mechanism that is dependent on the kinase activity of RAF1 but independent of the classical RAF1/MEK/ERK pathway, and suggest that RAF1 could target additional substrates in addition to MEK.

### 2.4. Discussion

LSA rapidly induces two main cellular phenotypic changes: cell contraction accompanied by plasma membrane blebbing (possibly through the activation of the actomyosin complex), and premature chromosome condensation independently of the classical mitosis promoting factor, cyclin B1/Cdk1 [12,13]. At the biochemical level, LSA induces the phosphorylation of a broad range of proteins associated with membrane, cytoskeleton and chromatin functions. These effects are rapid and reversible upon LSA removal, suggesting a direct impact of LSA on the activity of unidentified cellular kinase/phosphatases [12,13]. We recently reported that LSA is a relatively weak inhibitor of purified phosphatases 1 and 2A *in vitro* compared to okadaic acid [13]; thus, the molecular target(s) of LSA is still unclear, especially in intact cells, although it is likely to be a key regulator of chromatin and cytoskeleton organization.

**Figure 6:**
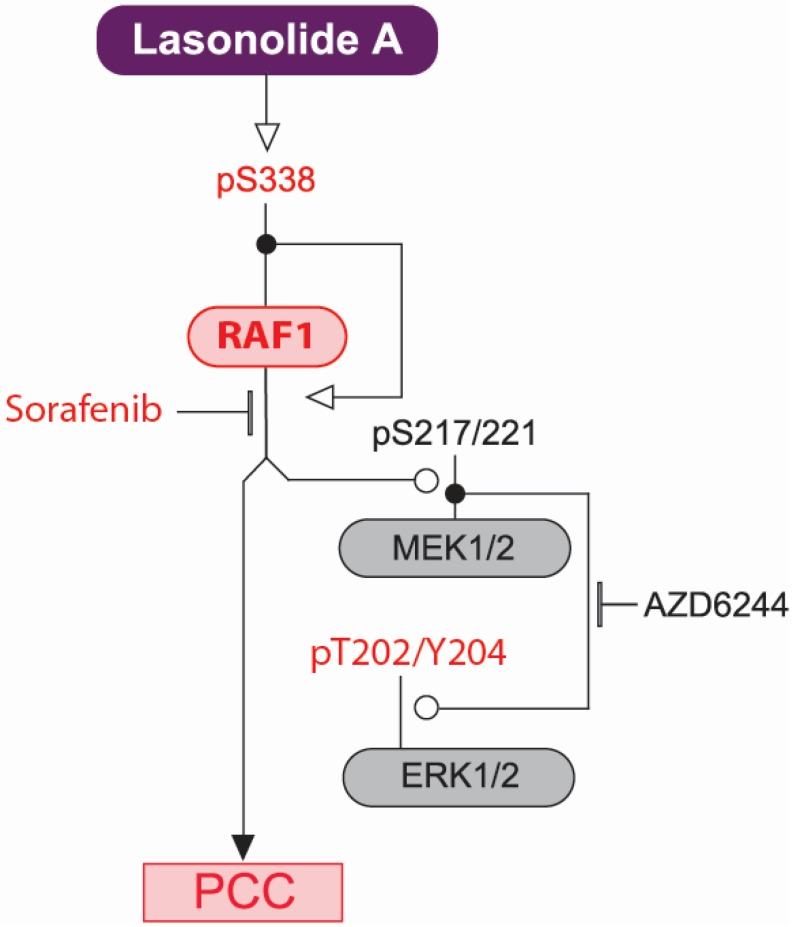
Proposed pathway of the major role of RAF1 in LSA-induced PCC. In red are the phosphorylation sites induced in response to LSA exposure. Annotations are derived from the Kohn molecular interaction map (MIM) conventions [38]. Nodes at intercepting lines are the post-translationally modified proteins. Open arrowhead indicates activation; open circles indicate phosphorylations, and short bars inhibition.

By screening a shRNA library targeting kinases and phosphatases we found several genes that are potentially involved in cellular sensitivity and resistance toward LSA. One unexpected mediator of LSA sensitivity was RAF1. Considering the major role of RAF1 and MAPK pathway in cell proliferation and oncogenesis, we focused on the activation of RAF1 by LSA. Three line of evidence implicate RAF1 in LSA-induced PCC. First, LSA induces the rapid phosphorylation of RAF1 on S338, a marker for activated RAF1 [26], in three cell cancer lines with different RAS status. As expected, activation of ERK1/2 was also observed in all three-cell lines, which is in agreement with independent results in Panc-1 cells [12]. Our findings therefore reveal that LSA activates RAF1 upstream of MEK/ERK. Second, we established a genetic link between RAF1 and LSA-induced PCC. Using both siRNA depletion of RAF1 in human cells and MEFs that are homozygous null for *Raf1*, we show that RAF1 is involved in LSA-induced PCC. Importantly, complementation experiment using WT and kinase dead RAF1 firmly established that RAF1 kinase activity is required for LSA-induced PCC. Of note, homozygous deletion of the *Braf* gene in MEFs did not prevent LSA-induced PCC; this experiment confirms that RAF1, but not BRAF, is required for LSA-induced PCC. Further supporting our genetic experiments, sorafenib, a RAF inhibitor [33], inhibited LSA-induced PCC. Unexpectedly, we found that the RAF1-mediated PCC induced by LSA is independent of the classical MAPK pathway, as it could not be blocked by the selective MEK inhibitor AZD6244. We conclude that LSA triggers chromatin condensation through RAF1 kinase independently of the MAPK pathway (Figure 6). Previous studies indicate that the mechanisms that activate RAF1 during mitosis are different from those that activate RAF1 following growth factor stimulation [18,19,35]. Further studies are warranted to determine the precise mechanism by which LSA stimulates the activation of RAF1. Of note, S338 of RAF1 has been reported as both an auto-phosphorylation site [20] and a site phosphorylated by PAK3 [36] and CaMK II [37]. In Raf1-null MEFs expressing kinase dead RAF1, LSA was able to induce S338 phosphorylation, therefore indicating a trans-phosphorylation mechanism is involved in RAF1 S338 phosphorylation. Further investigation is required to understand the regulation of RAF1 S338 phosphorylation. In which case, LSA could be a useful reagent, especially because the total synthesis of LSA has recently been improved [10,11].

We previously reported the impact of LSA on different key players of chromosome condensation and showed that LSA induces the activation of topoisomerase II and aurora kinases and the subsequent phosphorylation of histone H3. In addition, pharmacological inhibition of topoisomerase II or aurora kinases, but not CDK1, blocked LSA-induced PCC [13]. Recently, RAF1 has been implicated in mitotic progression independent of its kinase activity [35]. Our data, on the other hand, indicate that RAF1 kinase activity is essential in LSA-induced PCC. Thus further study is necessary to elucidate the mechanisms by which RAF1 mediates PCC.

In addition to PCC, we previous showed that LSA also induces actin cytoskeleton rearrangement and cell detachment. Several candidate hits from our shRNA screen, including MYPT1, PTP4A1 and TWF2, have all been implicated in regulating actin cytoskeleton dynamics [21,22,24]. RAF1 itself has been implicated in the regulation of cell motility and contractility through direct interactions with ROCK2 kinase and inhibiting its activity through a kinase-independent mechanism [39,40]. In LSA-treated cells, we observed both increase RAF1 phosphorylation and increase MYPT1 phosphorylation. The latter might be attributable to a potential role of LSA in inhibiting a MYPT1 phosphatase. Thus, further investigation is necessary to elucidate the mechanisms by which LSA affect the actin cytoskeleton.

LSA is a fascinating natural product due to its unique chemical structure and cellular activities. Our finding that LSA induces rapid RAF1 phosphorylation suggests that LSA might be useful as a tool to help identify RAF1 target proteins other than MEK and investigate the mechanism by which RAF1 regulates chromosome condensation.

## 3. Materials and Methods

### 3.1. Drugs and Antibodies

Lasonolide A was obtained by chemical synthesis as described [9]. AZD6244 and Sorafenib were obtained from DTP (Developmental Therapeutics Program, NIH). Antibodies against pS338-RAF1, RAF1 and pT696-MYPT1 were purchased from ThermoScientific, Santa Cruz and Upstate, respectively. GAPDH, pT202/Y204-ERK1/2 and pS19-MLC2 were from Cell Signaling.

### 3.2. Cell Culture and Treatments

The human cell lines, HCT-116, OVCAR-8 and CA46 were obtained from the Developmental Therapeutic Branch (DTP, National Cancer Institute, Rockville, MD, USA). MEFs WT, *Braf*^−/−^ and *Raf1*^−/−^ were provided by Manuela Baccarini. Cells were cultured in DMEM (HCT-116 and MEFs cells) or RPMI 1640 (OVCAR-8 and CA46 cells) medium supplemented with 10% fetal bovine serum. Cells at 60% confluency were used for LSA treatments.

### 3.3. shRNA Screening

The shRNA library and screening methodology was previously described [41]. The shRNA pool used contains 2951 shRNAs targeting most annotated human kinases and phosphatases (1140 RefSeqs). Retroviral shRNAs were delivered to cells at a multiplicity of infection ~1 and cells were selected for 3 days with puromycin for stable integration. Following selection, a fraction of cells were frozen down as starting samples and the rest were passaged in media with no LSA, 10 nM or 100 nM LSA. For each condition, 3 independent replicates were included. For treatment with high LSA (100 nM), cells were treated for 14 days without splitting and all surviving cells were collected. Cell counting indicated that cells have undergone the equivalent of 4 population doublings. For the control and low LSA (10 nM) treatment groups, cells were passaged when reaching ~80% of confluency. The control and low LSA populations underwent the equivalent of 17 and 10 population doublings, respectively, during the same 24-day period. A minimal representation of ~1000 was maintained for each replica during all stages of cell culture. shRNA sequences were PCR-recovered from genomic DNA of these cells and library composition in each sample was deconvolved by sequencing. To determine LSA-induced shRNA enrichment and dropout, the log2 ratios of normalized shRNA read counts between the end sample and the starting sample were calculated for each shRNA. The log2 ratios were then compared between the control (untreated) sample and the two LSA-treated arms to identify LSA-induced shRNA enrichment or dropout. Primary shRNA hits were identified as those with a log2 ratio difference of ≥1 and a *p* ≤ 0.01. STRING analysis was performed to identify protein-protein interactions among the top candidates. Relationships were mined using only experiment and database determined relationships of at least “medium confidence”.

### 3.4. Complementation of Raf^−/−^ MEFs

cDNA encoding wild-type-RAF1 and kinase dead (KD)-RAF1 where lysine 375 was mutated in alanine, were cloned downstream of the tetracycline response element in the pInducer 10b lentiviral vector. Plasmids were packaged using 293T cells with TransIT Transfection Reagent (Mirus Bio, Madison, WI, USA). *Raf1^−/−^* MEFs were transduced with pInducer WT-RAF1 and KD-RAF1 at a low MOI. Stably transduced cells were selected using 3 μg/mL puromycin for 3 days. Cells were tested with 1 μg/mL doxycycline for protein induction.

### 3.5. siRNA Transfection

Cells seeded in 6-well plates were transfected with targeting or non-targeting siRNAs using DharmaFECT transfection reagent (ThermoFisher Scientific, Waltham, MA, USA). Cells were exposed to LSA 72 h after transfection.

### 3.6. Protein Extracts and Western Blotting

Cell pellets were lysed with 1% SDS, 10 mM Tris-HCl, pH 7.4 with phosphatase inhibitor cocktail (Sigma-Aldrich, St Louis, MO, USA) and protease inhibitor (Roche Molecular Diagnostics, Mannheim, Germany). Total cell lysates were electrophoresed on 4%–20% Tris-glycine polyacrylamide gels, and transferred onto nitrocellulose membranes that were incubated with primary antibodies. After incubation with secondary antibody, signals were detected by enhanced chemiluminescence (Pierce, Rockford, IL, USA).

### 3.7. Chromosome Morphology

After drug treatment, cells were cytospun (800 rpm, 8 min) to glass slides and were fixed for 15 min with 4% paraformaldehyde in phosphate buffered saline (PBS, pH 7.4). After incubation for 20 min in 70% ethanol, the cells were stained with 20 μg/mL propidium iodide with 100 μg/ mL RNase A for 15 min in the dark. Finally, slides were mounted with Vectashield anti-fade mounting media (H-1000, Vector Laboratories, Inc., Burlingame, CA, USA). Images were taken using a Zeiss 780 confocal microscope (Carl Zeiss Inc., Thornwood, NY, USA).

### 3.8. Statistical Analyses

Data are presented as mean values ± SD. The significance of differences between means was assessed by Mann-Whitney test, with *p* < 0.05 and *p* < 0.01 considered statistically significant.

## 4. Conclusions

Lasonolide A (LSA) has unique and novel properties that warrant further studies and its evaluation as a novel anticancer agent. The activation of RAF1 and its association with rapid and reversible premature chromosome condensation by LSA reported here are remarkable and could serve as pharmacodynamics biomarkers together with histone phosphorylation and deacetylation, and topoisomerase II and MPM2 hyperactivation. Additional LSA-dependent kinase/phosphatase pathways were identified in our shRNA screen (PTP4A1, WNK1, PPP1R12A, TPK1, PK9L and PFKP), warranting further investigations. The recent improvement of synthetic routes will facilitate further mechanisms of action studies and the exploration of the anticancer activity of LSA in animal models.

## Supplementary Files

Supplementary File 1

## References

[1] Bailly C. (2004). Lamellarins, from A to Z: A family of anticancer marine pyrrole alkaloids. Curr. Med. Chem. Anticancer Agents.

[2] Newman D.J., Cragg G.M. (2014). Marine-sourced anti-cancer and cancer pain control agents in clinical and late preclinical development. Mar. Drugs.

[3] Mayer A.M., Glaser K.B., Cuevas C., Jacobs R.S., Kem W., Little R.D., McIntosh J.M., Newman D.J., Potts B.C., Shuster D.E. (2010). The odyssey of marine pharmaceuticals: A current pipeline perspective. Trends Pharmacol. Sci..

[4] Schumacher M., Kelkel M., Dicato M., Diederich M. (2011). Gold from the sea: Marine compounds as inhibitors of the hallmarks of cancer. Biotechnol. Adv..

[5] Radjasa O.K., Vaske Y.M., Navarro G., Vervoort H.C., Tenney K., Linington R.G., Crews P. (2011). Highlights of marine invertebrate-derived biosynthetic products: Their biomedical potential and possible production by microbial associants. Bioorg. Med. Chem..

[6] Pommier Y., Marchand C. (2012). Interfacial inhibitors: Targeting macromolecular complexes. Nat. Rev. Drug Discov..

[7] Changeux J.-P. (2011). 50th anniversary of the word “allosteric”. Protein Sci..

[8] Horton P.A., Koehn F.E., Longley R.E., McConnell O.J. (1994). Lasonolide A, a new cytotoxic macrolide from the marine sponge *Forcepia* sp.. J. Am. Chem. Soc..

[9] Ghosh A.K., Gong G. (2008). Total synthesis of potent antitumor agent (−)-lasonolide A: A cycloaddition-based strategy. Chem. Asian J..

[10] Ghosh A.K., Ren G.B. (2012). Stereoselective synthesis of both tetrahydropyran rings of the antitumor macrolide, (−)-lasonolide A. J. Org. Chem..

[11] Trost B.M., Stivala C.E., Hull K.L., Huang A., Fandrick D.R. (2014). A concise synthesis of (−)-lasonolide A. J. Am. Chem. Soc..

[12] Isbrucker R.A., Guzman E.A., Pitts T.P., Wright A.E. (2009). Early effects of lasonolide A on pancreatic cancer cells. J. Pharmacol. Exp. Ther..

[13] Zhang Y.W., Ghosh A.K., Pommier Y. (2012). Lasonolide A, a potent and reversible inducer of chromosome condensation. Cell Cycle.

[14] Gotoh E., Durante M. (2006). Chromosome condensation outside of mitosis: Mechanisms and new tools. J. Cell Physiol..

[15] Luo J., Emanuele M.J., Li D., Creighton C.J., Schlabach M.R., Westbrook T.F., Wong K.K., Elledge S.J. (2009). A genome-wide RNAi screen identifies multiple synthetic lethal interactions with the Ras oncogene. Cell.

[16] Mason C.S., Springer C.J., Cooper R.G., Superti-Furga G., Marshall C.J., Marais R. (1999). Serine and tyrosine phosphorylations cooperate in Raf-1, but not b-Raf activation. EMBO J..

[17] Dhillon A.S., von Kriegsheim A., Grindlay J., Kolch W. (2007). Phosphatase and feedback regulation of Raf-1 signaling. Cell Cycle.

[18] Laird A.D., Morrison D.K., Shalloway D. (1999). Characterization of Raf-1 activation in mitosis. J. Biol. Chem..

[19] Ziogas A., Lorenz I.C., Moelling K., Radziwill G. (1998). Mitotic Raf-1 is stimulated independently of ras and is active in the cytoplasm. J. Biol. Chem..

[20] Zang M., Gong J., Luo L., Zhou J., Xiang X., Huang W., Huang Q., Luo X., Olbrot M., Peng Y. (2008). Characterization of ser338 phosphorylation for Raf-1 activation. J. Biol. Chem..

[21] Achiwa H., Lazo J.S. (2007). Prl-1 tyrosine phosphatase regulates c-src levels, adherence, and invasion in human lung cancer cells. Cancer Res..

[22] Tu S.W., Bugde A., Luby-Phelps K., Cobb M.H. (2011). Wnk1 is required for mitosis and abscission. Proc. Natl. Acad. Sci. USA.

[23] Wang J., Kirby C.E., Herbst R. (2002). The tyrosine phosphatase prl-1 localizes to the endoplasmic reticulum and the mitotic spindle and is required for normal mitosis. J. Biol. Chem..

[24] Riento K., Ridley A.J. (2003). Rocks: Multifunctional kinases in cell behaviour. Nat. Rev. Mol. Cell Biol..

[25] Paavilainen V.O., Bertling E., Falck S., Lappalainen P. (2004). Regulation of cytoskeletal dynamics by actin-monomer-binding proteins. Trends Cell Biol..

[26] Matallanas D., Birtwistle M., Romano D., Zebisch A., Rauch J., von Kriegsheim A., Kolch W. (2011). Raf family kinases: Old dogs have learned new tricks. Genes Cancer.

[27] Abaan O.D., Polley E.C., Davis S.R., Zhu Y.J., Bilke S., Walker R.L., Pineda M., Gindin Y., Jiang Y., Reinhold W.C. (2013). The exomes of the nci-60 panel: A genomic resource for cancer biology and systems pharmacology. Cancer Res..

[28] Wu Q., Sahasrabudhe R.M., Luo L.Z., Lewis D.W., Gollin S.M., Saunders W.S. (2010). Deficiency in myosin light-chain phosphorylation causes cytokinesis failure and multipolarity in cancer cells. Oncogene.

[29] MacDonald J.A., Borman M.A., Muranyi A., Somlyo A.V., Hartshorne D.J., Haystead T.A. (2001). Identification of the endogenous smooth muscle myosin phosphatase-associated kinase. Proc. Natl. Acad. Sci. USA.

[30] Hu J., Stites E.C., Yu H., Germino E.A., Meharena H.S., Stork P.J., Kornev A.P., Taylor S.S., Shaw A.S. (2013). Allosteric activation of functionally asymmetric Raf kinase dimers. Cell.

[31] Freeman A.K., Ritt D.A., Morrison D.K. (2013). Effects of Raf dimerization and its inhibition on normal and disease-associated Raf signaling. Mol. Cell.

[32] Dent P., Reardon D.B., Morrison D.K., Sturgill T.W. (1995). Regulation of Raf-1 and Raf-1 mutants by Ras-dependent and Ras-independent mechanisms *in vitro*. Mol. Cell Biol..

[33] Beeram M., Patnaik A., Rowinsky E.K. (2005). Raf: A strategic target for therapeutic development against cancer. J. Clin. Oncol..

[34] Johannessen C.M., Boehm J.S., Kim S.Y., Thomas S.R., Wardwell L., Johnson L.A., Emery C.M., Stransky N., Cogdill A.P., Barretina J. (2010). Cot drives resistance to Raf inhibition through map kinase pathway reactivation. Nature.

[35] Mielgo A., Seguin L., Huang M., Camargo M.F., Anand S., Franovic A., Weis S.M., Advani S.J., Murphy E.A., Cheresh D.A. (2011). A mek-independent role for cRaf in mitosis and tumor progression. Nat. Med..

[36] King A.J., Sun H., Diaz B., Barnard D., Miao W., Bagrodia S., Marshall M.S. (1998). The protein kinase pak3 positively regulates Raf-1 activity through phosphorylation of serine 338. Nature.

[37] Salzano M., Rusciano M.R., Russo E., Bifulco M., Postiglione L., Vitale M. (2012). Calcium/calmodulin-dependent protein kinase II (CaMKII) phosphorylates Raf-1 at serine 338 and mediates Ras-stimulated Raf-1 activation. Cell Cycle.

[38] Kohn K.W., Aladjem M.I., Weinstein J.N., Pommier Y. (2006). Molecular interaction maps of bioregulatory networks: A general rubric for systems biology. Mol. Biol. Cell.

[39] Niault T., Sobczak I., Meissl K., Weitsman G., Piazzolla D., Maurer G., Kern F., Ehrenreiter K., Hamerl M., Moarefi I. (2009). From autoinhibition to inhibition in trans: The Raf-1 regulatory domain inhibits Rok-alpha kinase activity. J. Cell Biol..

[40] Ehrenreiter K., Piazzolla D., Velamoor V., Sobczak I., Small J.V., Takeda J., Leung T., Baccarini M. (2005). Raf-1 regulates rho signaling and cell migration. J. Cell Biol..

[41] Schlabach M.R., Luo J., Solimini N.L., Hu G., Xu Q., Li M.Z., Zhao Z., Smogorzewska A., Sowa M.E., Ang X.L. (2008). Cancer proliferation gene discovery through functional genomics. Science.

