# Supplementary material for: Activation of RAF1 (c-RAF) by the Marine Alkaloid Lasonolide A Induces Rapid Premature Chromosome Condensation
Source: Mar Drugs. 2015 Jun 5;13(6):3625–39. doi: 10.3390/md13063625 (PMC4483648; doi:10.3390/md13063625)
Supplement: Supplementary File 1 [file marinedrugs-13-03625-s001.pdf]

Supplementary Information

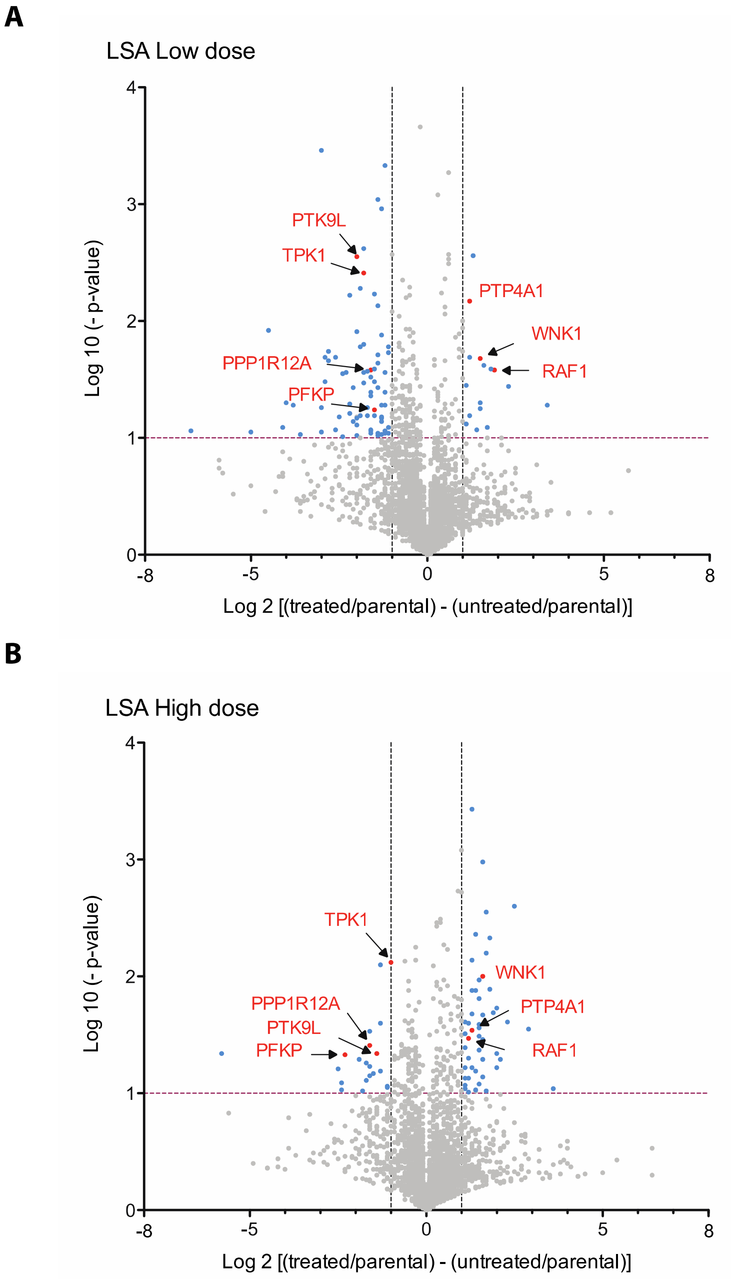

Figure S1. LSA synthetic lethal candidates. Volcano plot illustrating normalized shRNA fold changes (log base 2) and *p*-values (−log base 10) after cells were cultured in low, 10 nM (**A**) or high, 100 nM (**B**) LSA. Horizontal bar at $y = 1$ represents a significance level of $p = 0.1$ which we set as a relaxed cut-off. Vertical bars represents genes with a log 2 > 1 or < −1, *i.e.*, FC > 2 or < −2. Significantly modulated shRNAs by either LSA concentrations are colored in blue and commonly modulated shRNA by both LSA concentrations are colored in red.

© 2015 by the authors; licensee MDPI, Basel, Switzerland. This article is an open access article distributed under the terms and conditions of the Creative Commons Attribution license (http://creativecommons.org/licenses/by/4.0/).
